# Using Photovoice to Create Awareness and Proactive Attitudes Among Mothers of Preschool-Aged Children About the Prevalence of Ultraprocessed Food in the Local Food Environment

**DOI:** 10.5888/pcd23.250377

**Published:** 2026-03-12

**Authors:** Pamela Rothpletz-Puglia, Arthur Nabi, Ashley Hynes, Zainab Kutiyanawala, Thea Cogan-Drew, Nkechi Mbadugha, Veronica Jones

**Affiliations:** 1 Rutgers University School of Health Professions, New Brunswick, New Jersey; 2 The Leaguers, Inc, Health and Wellness, Newark, New Jersey

## Abstract

Ultraprocessed foods are deeply ingrained in US culture, and challenging the social acceptance of feeding children ultraprocessed foods is a priority. We used photovoice among a sample of mothers of preschool-aged children (n = 25) to assess their perceptions of the local food environment. Participants had 2 weeks before and after a nutrition education session to take photographs. Participants submitted 814 photographs of the local food environment, and mothers participated in 8 focus group discussions, each with 5 to 8 participants. After the education session, participants questioned the prevalence and marketing of ultraprocessed foods and discussed the need for change, thus signaling proactive attitudes.

**Summary**

**What is known about this topic?**

Ultraprocessed foods are deeply ingrained in US culture and encourage narrow diets with limited healthy options. Challenging the social acceptance of feeding children these foods is a priority.

**What is added by this report?**

We used photovoice to assess the perceptions of mothers of preschool-aged children of their local food environment. After taking photographs and participating in focus groups and a nutrition education session, mothers questioned the prevalence and marketing of ultraprocessed foods and discussed the need for change.

**What are the implications for public health practice?**

This project highlights the potential of participatory methods to generate grassroots momentum for advancing healthier food initiatives.

## Objective

Ultraprocessed foods are deeply ingrained in US culture and account for 58% to 65% of children’s energy intake (1–3). For example, common items on children’s menus, in media advertisements, and on grocery store promotions include chicken nuggets or tenders, hamburgers, grilled cheese, french fries, hot dogs, and macaroni and cheese (4). Foods like these are aggressively marketed and highly palatable, and they encourage narrow diets with limited healthy options. These factors, in turn, can create children’s resistance to new food, which can persist into adulthood, increasing lifetime risks of obesity and chronic disease (5).

Challenging the social acceptance of feeding children ultraprocessed foods is a priority, and raising awareness and reflection about their prevalence in the food environment is a crucial first step toward cultivating critical awareness (6). Photography involves active observation of the environment, making learning more personally relevant. Therefore, our partnership team, an academic institution, and a community agency collaborated to plan and implement a photovoice project. Photovoice is a research method in which community members identify and record issues in their communities by taking photographs, participating in dialogue about the problems, and disseminating the work to policymakers and other community members (7). This pilot study aimed to examine how participation in a photovoice project, combined with education on ultraprocessed foods, influenced mothers’ perceptions and evaluations of their local food environment.

## Methods

In alignment with Freire’s process of developing critical awareness (6), we used a community-based participatory research (CBPR) approach, using photovoice as the primary data collection method (6,8). After an introduction, participants conducted a food environment assessment using cameras, attended a nutrition education session, and then repeated the assessment. The educational session, held between the 2 assessments, focused on identifying ultraprocessed foods by examining ingredient lists and highlighting the benefits of unprocessed or minimally processed foods. The Rutgers University institutional review board approved the project.

The purposeful sample comprised female, English-speaking legal guardians (hereinafter “mothers”) of preschool-aged children who were African American or Black, biracial or multiracial, or Hispanic or non-Hispanic, representing the predominant racial and ethnic demographic characteristics of families served by the community agency. Mothers were included as participants because prior surveys showed that they are the main food procurers for families served by the community agency. The urban Head Start community agency recruited mothers through project flyers, parent discussions at drop-off, and parent meetings. Data collection consisted of photographs taken by participants, focus group discussions about the photographs, and an electronic demographic questionnaire. To facilitate the food environment assessment, we provided participants with a Kodak PIXPRO FZ45 digital camera (Eastman Kodak Company) and a secure digital card. Participants were instructed on how to use the camera and to conduct the assessment by photographing food present in their neighborhoods — such as that found in grocery stores, in restaurants, and at social activities — that influenced how they feed their children.

Participants had 2 weeks before and after the education session to take photographs. After the 2 weeks posteducation, 2 researchers trained in qualitative methods facilitated focus group sessions on a secure Zoom platform (Zoom Communications, Inc) and offered lunchtime, evening, and weekend options for convenience. The community agency partner recommended Zoom meetings over in-person meetings to meet the needs of busy parents. During the focus groups, mothers’ photographs were individually displayed for discussion (ie, photo elicitation) (9). These sessions lasted 60 to 75 minutes and were recorded and transcribed verbatim. The data were collected from February through May 2024. Participants received gift cards for their time.

We used SPSS version 29 (IBM) to analyze the questionnaire data and used NVivo version 14 (Lumivero) to analyze content from the focus group transcripts (10). Content analysis involved 2 analysts independently coding the focus group transcripts and photographs discussed. To ensure rigor, we conducted the data analysis after each focus group discussion to determine when we were no longer learning new information as an indicator for data saturation and implemented member checking during the photo-elicitation discussions to enhance credibility and objectivity (ie, participants clarified and verified interpretations). We held research team discussions to resolve discrepancies in coding between the analysts and to reach consensus on the analysis for dependability (11).

## Results

Thirty-three participants consented to the study; 25 mothers completed all components of the photovoice project and were included in the data analysis. Eight mothers missed sessions due to time or work constraints. Most participants were Black or African American (n = 23, 92%), and 2 mothers identified as 2 or more races (8%) (Table).

**Table:** Demographic Characteristics of Participating Mothers (n = 25), Photovoice Project on Ultraprocessed Food in the Local Food Environment, February–May 2024

Demographic characteristic	Number (%)
**Race**	
Black or African American	23 (92)
Two or more races	2 (8)
**Non-Hispanic ethnicity**	25 (100)
**Education**	
No high school diploma	2 (8)
GED or high school diploma	11 (44)
Some college	10 (40)
Completed bachelor’s degree or higher	2 (8)
**Relationship status**	
Married	4 (16)
Separated	3 (12)
Single	18 (72)

Abbreviation: GED, general educational development.

Participants submitted 814 photographs of their local food environment. Eight focus group discussions, each with 5 to 8 participants, were conducted, comprising 4 pre-education and 4 posteducation sessions. The key finding of the photovoice project was a shift from individual-level, family concerns to system-level critical awareness and proactive attitudes toward local food environment issues. The pre-education photograph discussions tended to focus on immediate family concerns, such as costs, time, and difficulties with shopping and feeding young children. In comparison, the posteducation photograph discussions focused more on critical awareness of external environmental influences in the food environment, such as advertising and the prevalence of ultraprocessed foods. For example, during a pre-education discussion, a mother presented photographs of ultraprocessed foods her children had chosen at the grocery store. During the post–photo-elicitation discussion, she said she began thinking about the items her children chose, so she used a wheelchair to see which foods were visible to her children at their height at the grocery store. She remarked that she was shocked by the marketing aimed at children. The Figure provides other examples of these shifts in conversations during the photo-elicitation discussions.

**Figure:**
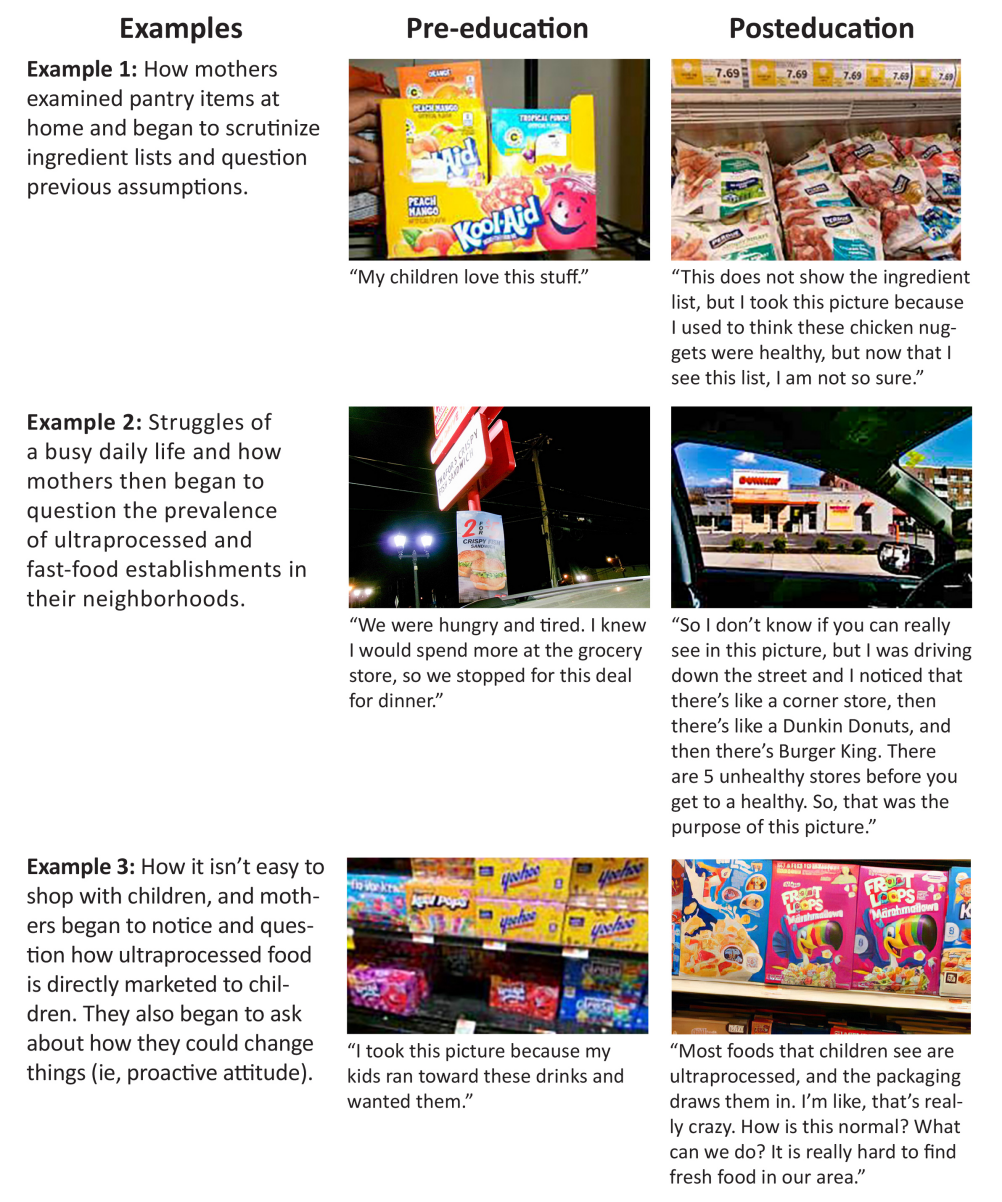
Examples of changes in photo-elicitation focus group discussions, pre-education and posteducation, among mothers of preschool-aged children, February–May 2024. The key finding of pre-education focused on immediate challenges related to family choices. The key finding of posteducation focused on an awareness of ultraprocessed food as well as more proactive attitudes about the system-level impacts on food for children.

## Discussion

The photovoice project facilitated perceptual shifts among mothers of preschool-aged children, from focusing on household-level struggles to recognizing the systemic influences shaping family food choices. The outcomes were similar to outcomes of other photovoice projects; the participating mothers began to question the status quo, reflecting the development of critical awareness and proactive attitudes (12–14). Although our project focused uniquely on developing critical awareness about the prevalence of ultraprocessed food in the food environment, a scoping review of 25 studies that used photovoice to understand overall access to healthy food demonstrated its effectiveness in nutrition research (15).

Critical awareness and proactive attitudes are essential for problem-oriented cognition, which influences behaviors and intentions to drive change (6,16). In accordance with CBPR practice, the participating mothers’ suggestions for change were disseminated in a photovoice exhibit at the community agency partner in December 2024 (8). Approximately 70 people attended, including academics, local leaders, community members, and representatives from various agencies. More information about the photovoice exhibit is available at https://sites.google.com/scarletmail.rutgers.edu/bps/kids-food-solutions.

Photovoice projects appear to be transformative, and the shift in perceptions of participating mothers was likely the result of active and collaborative learning opportunities provided through the approach, as well as the education (12–14). The educational session provided background information on ultraprocessed foods, and taking photographs engaged the mothers in observing their food environment as it relates to feeding young children. The focus group photo-elicitation discussions prompted reflection, leading to deeper understanding and connections, and enabled the documentation of the learning process. Participating in education and group discussions also may have influenced perceptions and subjective norms by providing social reinforcement and peer support, as described by Freire (6) and predicted by the Theory of Planned Behavior (17).

This was a small, observational, pilot study with inherent limitations, including limited data on actual behavior change or health outcomes. However, we used several strategies to ensure the rigor of the data analysis, and the quantity of photographs and the depth of discussions about them are indicators of the transferability of our findings to similar contexts.

Given that ultraprocessed foods are deeply ingrained in everyday life, it is notable that participants’ perceptions of them shifted (3). Participants questioned the prevalence and marketing of ultraprocessed foods and discussed the need for change, thus signaling proactive attitudes. Although further research is needed to evaluate this approach fully, this project highlights the promising potential of participatory methods to generate grassroots momentum for advancing healthier food initiatives.
